# Synergy at work: linking the metabolism of two lactic acid bacteria to achieve superior production of 2-butanol

**DOI:** 10.1186/s13068-020-01689-w

**Published:** 2020-03-11

**Authors:** Mette J. Mar, Joakim M. Andersen, Vijayalakshmi Kandasamy, Jianming Liu, Christian Solem, Peter R. Jensen

**Affiliations:** National Food Institute, Technical University of Denmark, Kemitorvet, Building 201, 2800 Kgs. Lyngby, Denmark

**Keywords:** 2-Butanol, *Lactococcus lactis*, Co-cultivation, *Lactobacillus brevis*, Diol dehydratase

## Abstract

**Background:**

The secondary alcohol 2-butanol has many important applications, e.g., as a solvent. Industrially, it is usually made by sulfuric acid-catalyzed hydration of butenes. Microbial production of 2-butanol has also been attempted, however, with little success as witnessed by the low titers and yields reported. Two important reasons for this, are the growth-hampering effect of 2-butanol on microorganisms, and challenges associated with one of the key enzymes involved in its production, namely diol dehydratase.

**Results:**

We attempt to link the metabolism of an engineered *Lactococcus lactis* strain, which possesses all enzyme activities required for fermentative production of 2-butanol from glucose, except for diol dehydratase, which acts on *meso*-2,3-butanediol (mBDO), with that of a *Lactobacillus brevis* strain which expresses a functional dehydratase natively. We demonstrate growth-coupled production of 2-butanol by the engineered *L. lactis* strain, when co-cultured with *L. brevis*. After fine-tuning the co-culture setup, a titer of 80 mM (5.9 g/L) 2-butanol, with a high yield of 0.58 mol/mol is achieved.

**Conclusions:**

Here, we demonstrate that it is possible to link the metabolism of two bacteria to achieve redox-balanced production of 2-butanol. Using a simple co-cultivation setup, we achieved the highest titer and yield from glucose in a single fermentation step ever reported. The data highlight the potential that lies in harnessing microbial synergies for producing valuable compounds.

## Background

Fermentative production of bio-ethanol is a classic example of microbial solutions for bio-based fuel production [1]. Ethanol, however, compared to medium length alcohols, such as butanol, has less desirable fuel properties [2]. Atsumi et al. successfully demonstrated the feasibility of producing different butanol isomers by coupling branched chain amino acid synthesis with the Ehrlich pathway [3], however, this approach is not applicable for producing 2-butanol and despite several attempts at its bio-production, so far only limited success has been reported.

Production of 2-butanol therefore relies on chemical synthesis, and currently 811,000 tons are being produced annually [4]. Besides its potential to serve as a biofuel, 2-butanol has numerous applications, e.g., as solvent or in perfume manufacturing [4].

Microbial production of 2-butanol from sugar has been achieved in *Klebsiella pneumonia* [5] albeit with low titers. Very recently, it was reported that 13.4 g/L 2-butanol could be produced from mBDO. The mBDO was generated by *Serratia marcescens* and subsequently converted into 2-butanol by *Lactobacillus diolivorans* [6]. There are clear limitations to using this approach, e.g., a very low yield of only 0.24 mol/mol glucose, and formation of large amounts of by-products such as acetate, ethanol, and lactate (in total 815 mM, 4.5 mol per mol 2-butanol). Furthermore, the need for a 30-min heat treatment to inactivate *S. marcescens*, and the use of this opportunistic pathogen for producing mBDO, appear not to be compatible with large-scale production of 2-butanol. Cell-free multi-enzyme catalysis has also been utilized for synthesis of 2-butanol from ethanol through continued supply of coenzyme B12 and ATP [7]. Additionally, 1.3 g/L butanone was made from glycerol through 3-ketovaleryl-CoA and subsequent decarboxylation [8], however, significant amounts of acetone was generated as by-product. Thus, there is room for further improvements in microbial 2-butanol production.

Production of 2-butanol in one-step fermentation setups typically involves the conversion of pyruvate into α-acetolactate, a reaction catalyzed by the α-acetolactate synthase. The α-acetolactate then undergoes decarboxylation into acetoin and reduction into mBDO. mBDO is subsequently dehydrated to 2-butanone followed by reduction 2-butanol.

Notably, the dehydration of mBDO to 2-butanone is carried out by the coenzyme B12-dependent diol or glycerol dehydratases [9], which are typically found in microorganisms capable of producing 1,3-propanediol [10]. B12-independent dehydratases have been described in *Clostridium butyricum*, however, these require an *S*-adenosyl methionine co-factor [11]. The coenzyme B12-dependent dehydratase reaction is oxygen sensitive and susceptible to irreversible inactivation when substrates such as glycerol and mBDO are used [12, 13]. To maintain catalytic activity, the microorganisms rely on dehydratase re-activation systems, consisting of reactivases, that consume ATP to restore catalytic activity [14]. The intracellular activity of the dehydratase is known to be influenced by several factors such as carbon source, growth phase, and the availability of inducer molecules [15].

Interestingly, the obligate heterofermentative *Lactobacillus brevis* was found to be capable of producing 2-butanol from the mBDO produced by yeast during wine fermentation [16]. Later, the diol dehydratases from *Lactobacillus brevis* were found to be superior to dehydratases from *Klebsiella oxytoca* and *Salmonella enterica* [17]. Lactic acid bacteria (LAB), best known for their application in dairy fermentations and as human probiotics, have recently been demonstrated to have great potential for use in biotechnological applications [18]. The emergence of tools for genetic engineering of LAB [19], combined with their high metabolic rates and fast growth [20], make them interesting candidates for production of biofuels. One particular LAB, *Lactococcus lactis*, has received a lot of attention, and has been metabolically engineered into producing a broad variety of useful compounds [21].

In our previous work, we constructed an *L. lactis* strain that could be used as a platform for producing various pyruvate-derived compounds, with little by-product formation [22]. Recently, we expanded the metabolic repertoire of this strain by introducing genes needed for production of mBDO [23], the precursor for 2-butanol.

In the current study, we first investigate whether *L. lactis* is the right platform for producing 2-butanol and we do this by introducing a diol dehydratase from *Klebsiella oxytoca* and a 2-butanol dehydrogenase from *Achromobacter xylosoxidans*. Challenges in achieving a functional diol dehydratase prompt us to try out a different strategy, namely co-cultivation, where we explore whether the diol dehydratase of *L. brevis* can complement an incomplete 2-butanol biosynthetic pathway in an engineered *L. lactis* strain. We show that co-cultivation is an efficient approach for producing 2-butanol, and achieve the highest reported titer and yield from glucose in a one-step fermentation process.

## Results and discussion

### Assessing the potential of *L. lactis* for 2-butanol production

**Fig. 1:**
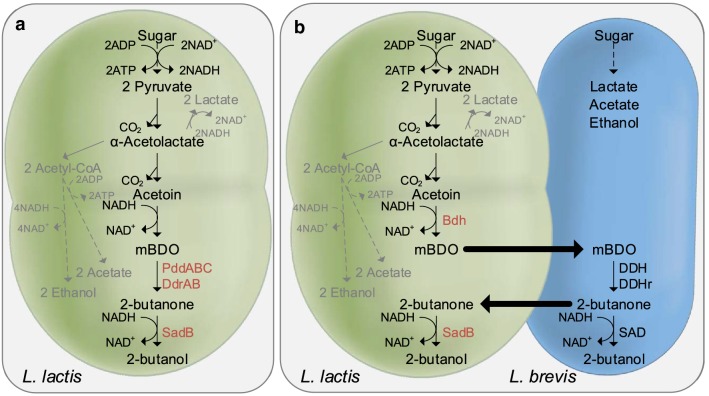
Linking the metabolism of *L. lactis* and *L. brevis* to achieve 2-butanol production. **a** Metabolic pathway based on *L. lactis* CS4363 (mBDO added in the medium). The constructed strain encodes: PddABC, diol dehydratase, and DdrAB, reactivase from *K. oxytoca*; SadB, secondary alcohol dehydrogenase from *A. xylosoxidans*. Pathways in gray indicate activities that have been eliminated. Dashed lines indicate multiple enzymatic steps. **b** The combined metabolic pathway for *L. lactis* and *L. brevis*. Bdh, butanediol dehydrogenase from *Enterobacter cloacae.* DDH, diol dehydratase; DDHr, diol dehydratase reactivate; SAD, secondary alcohol dehydrogenase. Only heterologously expressed gene activities in *L. lactis* and activities related to 2-butanol synthesis in *L. brevis* are highlighted

*Lactococcus lactis* is an established industrial workhorse within the dairy industry, where it is used to ferment in excess of 100 mio. tonnes of milk annually [24]. This lactic acid bacterium grows well, is easy to manipulate genetically [25–27] and there are many reports on its use as an efficient cell factory for producing useful compounds [21, 23, 28, 29]. Here we explore whether *L. lactis* can be transformed into a 2-butanol-producing cell factory. To assess the potential of *L. lactis* to become an efficient 2-butanol producer, we first introduced two genes necessary for 2-butanol formation from mBDO, namely a diol dehydratase for converting BDO into 2-butanone, and an alcohol dehydrogenase for reducing 2-butanone into 2-butanol. We used the *L. lactis* strain CS4363, which lacks lactate dehydrogenase, phosphotransacetylase, and alcohol dehydrogenase activities, and can only grow under aerated conditions where NADH oxidase regenerates NAD^+^ and its sole fermentation product is acetoin. By introducing the diol dehydratase and alcohol dehydrogenase enzyme activities into CS4363, redox-balanced production of 2-butanol from mBDO should in principle be possible (Fig. 1a). For the diol dehydratase, we decided to rely on the enzyme complex from *K. oxytoca* (PddABC), and the alcohol dehydrogenase was obtained from *A. xylosoxidans* (SadB). SadB has previously been found to be efficient at converting 2-butanone into 2-butanol [30] and the diol dehydratase from *K. oxytoca* has previously been demonstrated to be efficient at dehydrating mBDO [17]. One concern when using diol dehydratases for dehydrating mBDO, is substrate inactivation, and the enzyme needs to be re-activated by a dedicated reactivase. For this reason we additionally introduced the diol dehydratase reactivase from *K. oxytoca* (DdrAB), as the beneficial effect of this has been demonstrated previously [31].

After introducing the genes, we verified the respective enzyme activities. We found that the recombinant strain, in contrast to its parent lacking 2-butanol dehydrogenase activity (SadB), could grow anaerobically in the presence of 2-butanone with concurrent formation of 2-butanol, which confirmed the presence of SadB activity. The diol dehydratase activity was measured in crude cell extracts and was shown to be 0.32 ± 0.01 µmol min^−1^ mg protein^−1^. We subsequently examined if the engineered strain could grow and produce 2-butanol from mBDO in medium containing coenzyme B12, a co-factor needed for the function of the diol dehydratase. However, we did not observe restoration of anaerobic growth or formation of 2-butanol when mBDO was supplied. *L. lactis* lacks genes involved in coenzyme B12 biosynthesis, and our findings that coenzyme B12 is not taken up by the intact cells, is in accordance with the absence of an uptake system for vitamin B12 in *L. lactis* [32].

### *Lactobacillus brevis* can serve as a whole-cell diol dehydratase catalyst

The observation above strongly indicates that the reason why our engineered *L. lactis* strain cannot produce 2-butanol is due to low or no diol dehydratase activity resulting from the lack of coenzyme B12 uptake. In principle, we could pursue heterologous introduction of genes involved in B12 synthesis from organisms possessing these, e.g., *Lactobacillus reuteri* [33], but the B12 synthesis pathway is encoded by 29 genes [34]. As an alternative, we decided to explore whether the diol dehydratase activity could be supplied, in-trans, from a second strain, used as a whole-cell catalyst. In the subsequent experiments we chose to express the *Enterobacter cloacae* meso-2,3-butanediol dehydrogenase (Bdh) and the 2-butanol dehydrogenase (SadB) in *L. lactis*, thus generating a strain which in principle only lacks a diol dehydratase in order to be able to generate 2-butanol (Fig. 1b). As a source of the diol dehydratase we chose *L. brevis* SE20, which previously has been shown to produce 2-butanol when supplied with mBDO and vitamin B12 [35].

Our hypothesis was that the mBDO formed in *L. lactis* would leave the cells and enter the *L. brevis* cells to be dehydrated into 2-butanone. 2-butanone would subsequently leave the *L. brevis* cells, reenter the *L. lactis* cells and be reduced into 2-butanol. In this way, the metabolism of *L. lactis* would be redox balanced, since the two NADH generated in glycolysis would be consumed by the 2,3-butanediol dehydrogenase and the 2-butanol dehydrogenase.

We found that glucose was a poor substrate for *L. brevis*, probably due to a low ATP yield on glucose of only one [36]. On xylose, however, the ATP yield is two (see Additional file 1: Figure S1). Using xylose as a fermentation substrate could be of interest, since this sugar is abundant in lignocellulose. However, since we intend to use *L. brevis* as an mBDO dehydratase cell catalyst, it is relevant to investigate on which substrate the highest in vivo enzyme activity is attained. We found a ninefold higher mBDO dehydratase activity for cells grown on glucose when compared to cells grown on xylose, and that mBDO acted as an inducer of activity (Table 1). For the following experiments, we therefore decided to rely on *L. brevis* cells grown on glucose.

**Table 1:** In vivo mBDO dehydratase activity of *L. brevis* on different carbon sources

Carbon source ± inducer	Activity (U/OD_600_)
Glucose	8.6 ± 0.6
Glucose + mBDO	10.4 ± 0.9
Xylose	1.0 ± 0.03
Xylose + mBDO	4.0 ± 0.4

Values are average of three independent measurements with standard deviations

The next step was to test if the diol dehydratase activity from *L. brevis* could complement the metabolism of the engineered *L. lactis*, and thereby enable production of 2-butanol by *L. lactis*. Indeed, 2-butanol synthesis was achieved in defined medium (SA) supplemented with 7.5 µM vitamin B12 and 5 mM 2-butanone. After 20 h, a titer of 14.2 ± 0.6 mM, with a yield of 0.5 ± 0.02 mol/mol was obtained. Production of 2-butanol was not observed when the cultures were incubated in medium without a small “catalytic” amount of 2-butanone added, which we speculate helped in the linking of the metabolisms of the two bacteria. We also demonstrated that 2-butanol production could be accomplished using a mix of lactose and xylose, although with a lower titer and yield (see Additional file 1: Table S1).

### Co-cultivation of engineered *L. lactis* and *L. brevis* in M17 broth

After demonstrating proof-of-principle, we established a fermentation setup for co-culturing the two strains to enable more efficient 2-butanol production. The aim was to create an environment supporting a high metabolic flux in *L. lactis*, thus enabling efficient 2-butanol production, while concurrently preserving a high dehydratase activity in *L. brevis*. For the latter, an active *L. brevis* metabolism is needed, as re-activation of the diol dehydratase requires ATP. We decided to use rich M17 medium supplemented with 2% glucose, which supports optimal growth of *L. lactis* and to this medium 7.5 µM B12 was added. It has been shown previously that the ratio between the different strains present in a co-culture has a great impact on product formation [37]. For this reason, three different inoculation ratios of *L. lactis* to *L. brevis* were tested, 1:1, 1:4, and 4:1, using cell densities corresponding to an OD_600_ of either 0.06 or 0.24.

**Fig. 2:**
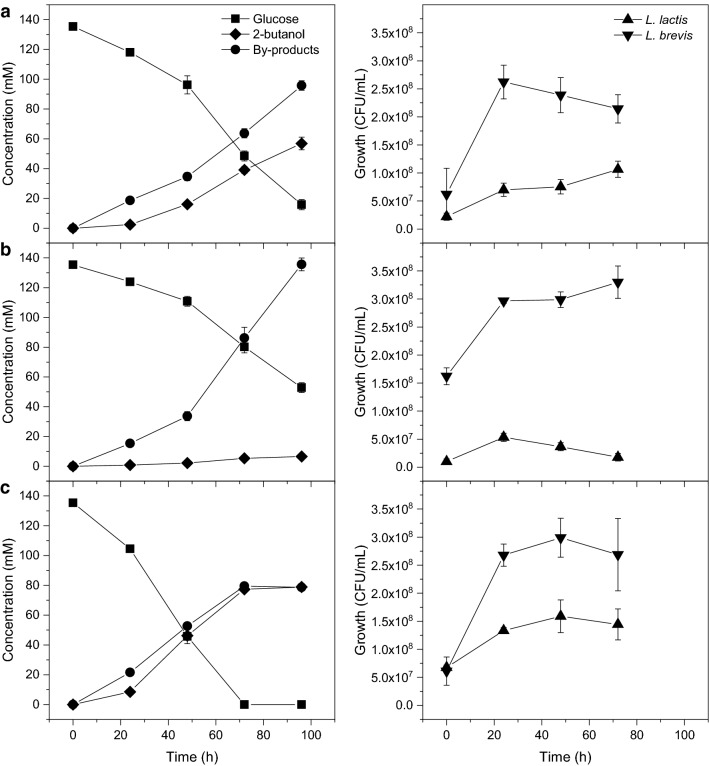
Product formation (left) and growth (right) for co-cultivation of recombinant *L. lactis* and *L. brevis*. Inoculation ratios *L. lactis*:*L. brevis* of 1:1, 1:4, and 4:1, **a**, **b**, and **c**, respectively. Average of three independent experiments with standard deviations

We found that 2-butanol was formed, when using M17 medium as well (Fig. 2 and Table 2). When using M17 medium, it was not necessary to add 2-butanone to facilitate 2-butanol generation. The best performance was observed when an excess of *L. lactis* was used (inoculation ratio 4:1), with a production of 80.0 ± 1.0 mM (5.9 ± 0.1 g/L) 2-butanol and a yield of 0.58 ± 0.01 mol/mol.

**Table 2:** Co-culture fermentation yield of products after 96 h at different ratios of inoculation

*L. lactis*:*L. brevis*	Glucose cons. (mM)^a^	2-Butanol **(**mol/mol glucose)	2-Butanone	By-products^b^
1:1	120 ± 3.4	0.48 ± 0.02	0.03 ± 0.01	0.80 ± 0.05
1:4	82.6 ± 3.3	0.08 ± 0.01	0.02 ± 0.001	1.64 ± 0.02
4:1	135 ± 3.4	0.58 ± 0.01	0.04 ± 0.01	0.58 ± 0.01

Yield was calculated from the fermentation experiment shown in Fig. 2. Average of three independent experiments with standard deviations

*ND* not detected

^a^Glucose consumed

^b^By-products, sum of acetate, ethanol, and lactate

The 4:1 culture also resulted in the lowest production of the by-products acetate, ethanol, and lactate. None of the co-cultivations showed significant buildup of 2-butanol precursors, which suggests an effective transfer of intermediates between the two strains. 2-Butanol was not produced in any of the control cultivations with *L. lactis* or *L. brevis* alone, and only modest glucose consumption was observed in these cultures (data not shown). Additionally, growth of the *L. lactis* strain was dependent on the catalytic activity of the *L. brevis* strain.

Formation of the by-products acetate, ethanol, and lactate during co-cultivation was from 79 to 136 mM, as compared to *L. brevis* alone where 56 ± 1.5 mM was produced. The increase in by-product formation observed in the co-cultures suggests that *L. brevis*, in addition to catalyzing the conversion of mBDO to 2-butanone, reduce some of the 2-butanone to 2-butanol. This issue becomes more pronounced at higher initial culture ratios where the lack of 2-butanone in combination with the high acid production by *L. brevis* begins to inhibit *L. lactis*, which then reaches lower CFU/mL.

**Fig. 3:**
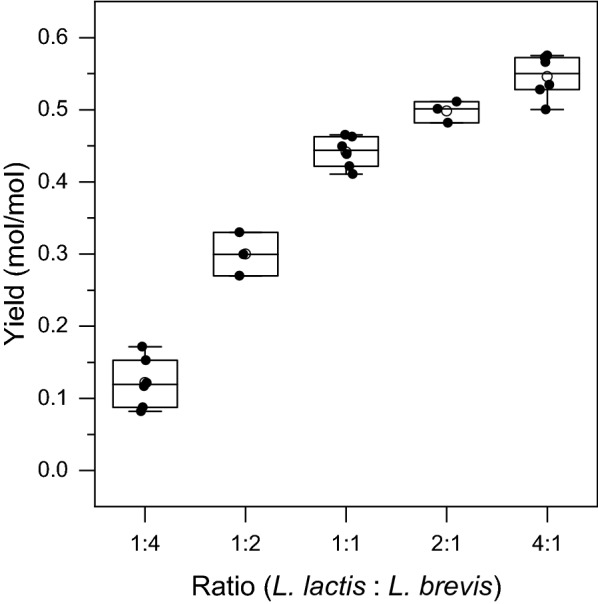
Co-culture yield of 2-butanol at different inoculation ratio of *L. lactis* and *L. brevis*. Production after 72 h of cultivation. Average of 3 or 6 independent experiments

It therefore appears to be important to restrict the amount of *L. brevis* cells present to avoid excessive consumption of 2-butanone, while simultaneously ensuring that a sufficient diol dehydratase activity is available. We tested other inoculation ratios as well, however, this did not lead to higher yields of 2-butanol (Fig. 3). Previous research into co-culture fermentations highlights division of labor and functional enzyme expression to be the main burden of monocultures, whereas co-culturing is constrained by the need of population control and possible limitation by transfer of intermediates [38, 39].

We believe that there is potential for improving the titer and yield of 2-butanol by further engineering of the strains and by optimizing the fermentation setup. In the setup used here, we relied on a wild-type *L. brevis* strain to supply the important diol dehydratase activity. Protein engineering has been used to improve the diol dehydratase performance [40], and when the improved enzyme was introduced into *K. pneumoniae*, this resulted in improved 2-butanol production [5]. It has also been reported that over-expression of the transcription factor PocR in *L. brevis* can boost the diol dehydratase activity of *L. brevis* [41].

## Conclusion

Our work highlights the possibility of linking the metabolisms of living microorganisms for producing useful compounds. Here, we have used an engineered *L. lactis* and a wild-type *L. brevis* strain for producing 2-butanol, where the *L. lactis* strain depends on the diol dehydratase activity of the *L. brevis* strain. We achieved the highest titer (5.9 g/L) and yield (0.58 mol/mol glucose) ever reported in a one-step production setup, and we believe that our work sets the stage for future studies where metabolisms of microorganisms are linked to enable superior production of a variety of useful compounds.

## Methods

### Strains and plasmids

Strain construction in *L. lactis* was based on MG1363, a plasmid-free derivative of *L. lactis* subsp. *cremoris* strain NCD0712 [42]. For optimized production of the precursor acetoin, CS4363 (MG1363 *Δ*^*3*^*ldh Δpta ΔadhE*) was used [22]. Expression of heterogeneous genes in *L. lactis* was done using plasmids pCI372 and pTD6. Derivatives of MG1363 and plasmids used in this study are described in Table 3.

**Table 3:** *Lactococcus lactis* strains and plasmids used in this study

Designation	Genotype or description	References
Strains		
CS4363	MG1363 Δ^3^*ldh* Δ*pta* Δ*adhE*	[22]
MM01	CS4363 pButop pDdrAB	This work
MM10	CS4363 pButop	This work
MM06	CS4363 pMM06	This work
Plasmids		
pTD6	*E. coli*/*L. lactis* shuttle vector containing gusA reporter, Tet	[22]
pJM001	pTD6::*bdh*, Tet	[43]
pCI372	*E. coli*/*L. lactis* shuttle vector, Cam	[44]
pButop	pCI372::*pddABC*-*sadB*	This work
pDdrAB	pTD6::ddrAB	This work
pMM06	pTD6::*bdh*-*sadB*	This work

*Lactobacillus brevis* SE20 [35], isolated from an ethanol pilot plant facility in Örnsköldsvik Sweden was kindly provided by Christer Larsson (Chalmers University of Technology, Sweden). *Escherichia coli* strain Top10 {F-*mcr*A Δ(*mrr*-*hsd*RMS-*mcr*BC) φ80*lac*ZΔM15 Δ*lac*Χ74 *rec*A1 *ara*D139 Δ(*ara*-*leu*) 7697 *gal*U *gal*K *rps*L (StrR) *end*A1 *nup*G λ-} was used for cloning purposes.

### Growth conditions

Cultivation of *L. lactis* and *L. brevis* were carried out in 125-mL flasks with 100 mL medium and slow magnetic stirring at 30 °C.

For growth experiments, *L. lactis* was cultivated in M17 medium (Oxoid, England) or defined synthetic amino acid (SA) medium [45] with the following modification: 40 mM MOPS was replaced with 100 mM potassium phosphate buffer. Both media were supplemented with 1% glucose. For test of activity of the expressed diol dehydratase and alcohol dehydrogenase, cultivations were executed in M17 medium with 7.5 µM coenzyme B12 and 20 mM mBDO or 2-butanone. Strains unable to grow anaerobically were cultivated aerobically.

*Lactobacillus brevis* was grown in modified MRS medium [46] containing per liter: peptone, 10 g; meat extract, 10 g; yeast extract, 5 g; Tween 80, 1 mL; K_2_HPO_4_, 2 g; sodium acetate·3H_2_O, 5 g; triammonium citrate, 2 g; MgSO_4_·7H_2_O, 0.2 g; MnSO_4_·4H_2_O, 0.05 g; glucose or xylose, 20 g, 7.5 µmol vB12. When needed, 20 mM mBDO was added to stimulate expression of diol dehydratase.

*Escherichia coli* strains were grown aerobically at 37 °C in Luria–Bertani broth [47].

When required, antibiotics were added in the following concentrations: tetracycline, 8 µg/mL for *E. coli* and 5 µg/mL for *L. lactis*; chloramphenicol, 20 µg/mL for *E. coli* and 5 µg/mL for *L. lactis.*

### DNA techniques

All manipulations were performed according to Sambrook and Russell [47]. *E. coli* was transformed using electroporation. *L. lactis* was made electrocompetent by growing in GM17 medium containing 1% glycine and transformed by electroporation as previously described by Holo and Nes, 1989 [48]. Chromosomal DNA from *L. lactis* was isolated using the method described for *E. coli* by Sambrook and Russel [47] with the modification that cells were treated with 20 μg of lysozyme per mL for 2 h prior to lysis.

### Construction of strains

For construction of a 2-butanol-producing *L. lactis*, the diol dehydratase and reactivase from *K. oxytoca* ATCC 8724 [49] and 2-butanol dehydrogenase from *A. xylosoxidans* [30] were codon-optimized for *L. lactis* and synthesized by Genscript. p*ddABC* and *sadB* and GapB promotor from *L. lactis* was amplified using the primers VP19 (SalI) and VP20 (PstI) (Table 4). The PCR products were further cloned into the XbaI/KpnI and PstI/SalI sites of pCI372, resulting in plasmid pButop. The plasmid was further transformed into strain CS4363 (MG1363 Δ^3^*ldh* Δ*pta* Δ*adhE*), a plasmid-free derivative of *L. lactis* subsp. *cremoris* strain NCD0712 [42], resulting in strain MM10. *ddrAB* and GapB promotor from *L. lactis* was amplified using primers P001 and P002 and cloned at SalI/PstI of pTD6. The plasmid was transformed into MM10, resulting in strain MM01.

**Table 4:** Primers used in this study

Primer name	Primer use	Primer sequence (5′→3′)
VP20	GapB promotor, PstI	ATCACTGCAGGAATAAAAATTACTGACAGC
VP19	GapB promotor, SalI	TATCAGTCGACTAGTAGTTTCCTCCTTATAG
P001	*ddrAB* + gapB, ups., PstI	ACGCCTGCAGGAATAAAAATTACTGACAGCC
P002	*ddrAB*, dwn., SalI	TGCGGTCGACTTATTCATCTTGTTGTTCACC
P038	*sadB* + gapB, ups., gibson	CCCTATAAGGAGGAAACTACTAATGAAAGCATTAGTATATCATGGAG
P039	*sadB*, dwn., gibson	AATTCTGTGTTGCGCATGCGGGTACCTTATGCTGCTCCT
P041	pJM001, gibson	TCGAGCTCCATGGCATATG
P036	pJM001, gibson	TAGTAGTTTCCTCCTTATAGGGATTAGTTAATTAAATACCATACCACCATCA

For application in co-cultivation, construction of a vector for high production of the precursor mBDO and expression of the 2-butanol dehydrogenase, *sadB,* was based on plasmid pJM001 [43]. pJM001 encode a codon-optimized butanediol dehydrogenase from *E. cloacae*, *bdh*. Plasmid pMM06 was constructed using Gibson assembly of *sadB* amplified using primers P038 and P039 and pJM001 amplified using primers P041 and P036. The plasmid was further transformed into *L. lactis* strain CS4363 to generate MM06.

### Analytical methods

Cell growth was regularly monitored by measuring optical density at 600 nm (OD_600_) and the quantification of glucose, xylose, lactate, acetate, acetoin, ethanol, mBDO, 2-butanone, and 2-butanol was carried out using an Ultimate 3000 high-pressure liquid chromatography system (Dionex, Sunnyvale, USA) equipped with a Aminex HPX-87H column (Bio-Rad, Hercules, USA) and a Shodex RI-101 detector (Showa Denko K.K., Tokyo, Japan). The column oven temperature was set at 60 °C and the mobile phase consisted of 5 mM H_2_SO_4_, at a flow rate of 0.5 mL/min.

### Assays

Diol dehydratase activity of MM10 towards 1,2-propanediol (PDO) was determined in cellular extracts using the 3-methyl-2-benzothiazolinone hydrazone (MBTH) method [50]. MBTH reacts with the produced propionaldehyde to form an azine derivate which can be determined by spectrophotometer [51]. Cells from a 100-mL culture were harvested, washed twice, and re-suspended in 10 mM potassium phosphate and 1 mM dithiothreitol buffer, pH 7.2. The cells were then disrupted by glass beads (106 µm, Sigma, Prod. No. G4649) using a FastPrep (MP Biomedicals, Santa Ana, USA). The reaction of 0.5 mL contained 50 mM potassium chloride, 35 mM potassium phosphate buffer pH 8, 0.015 mM coenzyme B12, 50 mM PDO, and appropriate amount of cellular extract. After incubation at 37 °C for 10 min, the reaction was terminated by addition of 0.5 mL potassium citrate buffer (0.1 M pH 3.6). 0.25 mL 0.5% MBTH hydrochloride was added, left to react at 37 °C after 15 min 0.5 mL water was added prior measurement at 305 nm using the Infinite M1000 PRO microplate reader. Absorbance values were converted to µmol propionaldehyde using standard curve. Protein concentration of cellular extracts was determined using the Bradford method, and bovine serum albumin served as the standard.

mBDO dehydratase activity was determined in vivo in cells of SE20 cultivated in modified MRS medium with 2% glucose or xylose, with or without addition of 20 mM mBDO. Cultures were harvested at late exponential phase, washed with 0.9% sodium chloride, and re-suspended to OD_600_ of 2.5 for conversion of 20 mM mBDO. Incubations were executed at 30 °C in SA medium added 7.5 µM vB12 and 1% glucose or xylose. Product formation was determined as the sum of 2-butanone and 2-butanol produced after 3 h of incubation.

### Co-cultivation in SA medium

For co-cultivations, the strains were pre-cultivated separately to obtain biomass, harvested at late exponential phase by centrifugation (5000*g*, 10 min), and re-suspended in co-cultivation medium at the desired inoculum OD_600_. Pre-cultivation of *L. lactis* strains was done in SA medium with glucose, tetracycline and 20 mM 2-butanone to sustain anaerobic growth. *L. brevis* cultivations were done in modified MRS medium containing 2% glucose and 20 mM mBDO.

Co-cultivations were done in SA medium with 2% glucose, 7.5 µM vB12 and 5 mM 2-butanone. Inoculations were done in start OD_600_ of 1:1, 1:0, and 0:1, of *L. lactis* to *L. brevis*. Product formation was determined after 20 h of incubation. Cultures were prepared as biological triplicates.

### Co-cultivation in M17 medium

For co-cultivations *L. lactis* MM06 and *L. brevis* SE20 were grown separately and harvested by centrifugation at late exponential phase (OD_600_ = 0.7 and OD_600_ = 0.4, respectively). *L. lactis* was cultivated in M17 supplemented with 1% glucose, tetracycline and 10 mM 2-butanone. *L. brevis* was cultivated in modified MRS with 1% glucose and supplemented with 20 mM mBDO.

Co-cultivations were executed in M17 with 2% glucose and 7.5 µM vB12. Minimal stirring was applied to the 50-mL tubes using 1 cm rod-shaped stirring magnets to keep the culture turbid while avoiding aeration. Cultures were inoculated to a final OD_600_ value of either 0.06 or 0.24 for each strain resulting in combinations of *L. lactis*:*L. brevis* of 1:1, 1:4, 4:1, 1:0, and 0:1. Cultures were incubated for 96 h and samples taken every 24 h for OD_600_, HPLC, and CFU analysis. Cultures were prepared as biological triplicates. To verify batch-to-batch replicability, an additional co-cultivation was executed using inoculation ratios of *L. lactis*:*L. brevis* of 1:4, 1:2, 1:1, 2:1, and 4:1. Product formation was evaluated after 72 h of cultivation.

Determination of colony forming units (CFU) during co-cultivation of *L. lactis* MM06 and *L. brevis* SE20 were done on agar plates consisting of a semi-defined medium [52] supplemented with 1.5% agar (w/v), 1% glucose (w/v) and 200 µM X-gluc (5-bromo-4-chloro-3-indolyl-beta-d-glucuronic acid) for colorimetric detection of β-glucuronidase activity. On these plates, the *L. lactis* appear as large blue colonies, whereas *L. brevis* appear as small white colonies.

## Supplementary information

**Additional file 1: Figure S1.** Overview of glucose and xylose metabolism in *L. brevis*. On glucose, two NADH are formed in the oxidative pentose phosphate pathway, and these have to be oxidized through ethanol formation from acetyl-CoA. Thus, the Acetyl-P cannot give rise to ATP formation through the action of acetate kinase. On xylose, however, there is no such constraint, and the acetyl-P can be used for generating ATP. **Table S1.** Production of 2-butanol from lactose and xylose in defined SA medium using resting cells of *L. lactis* and *L. brevis*.

## Data Availability

All data generated and analyzed during the current study are included in this published article and its supplementary information file.

## Abbreviations

*L. lactis*: *Lactococcus lactis*
*L. brevis*: *Lactobacillus brevis*
*S. marcescens*: *Serratia marcescens*
*K. oxytoca*: *Klebsiella oxytoca*
*A. xylosoxidans*: *Achromobacter xylosoxidans*
*E. cloacae*: *Enterobacter cloacae*
mBDO: *meso*-2,3-Butanediol
LAB: Lactic acid bacteria
NADH or NAD^+^: Reduced or oxidized form of nicotinamide adenine dinucleotide
PddABC: Diol dehydratase from *K. oxytoca*
SadB: Alcohol dehydrogenase from *A. xylosoxidans*
DdrAB: Diol dehydratase reactivase from *K. oxytoca*
Bdh: Butanediol dehydrogenase from *E. cloacae*
OD_600_: Optical density at wavelength 600 nm
SA: Synthetic amino acid medium
