# Supplementary material for: Synergy at work: linking the metabolism of two lactic acid bacteria to achieve superior production of 2-butanol
Source: Biotechnol Biofuels. 2020 Mar 11;13:45. doi: 10.1186/s13068-020-01689-w (PMC7065357; doi:10.1186/s13068-020-01689-w)
Supplement: Supplementary file 1 — Additional file 1: Figure S1. Overview of glucose and xylose metabolism in L. brevis. On glucose, two NADH are formed in the oxidative pentose phosphate pathway, and these have to be oxidized through ethanol formation from acetyl-CoA. Thus, the Acetyl-P cannot give rise to ATP formation through the action of acetate kinase. On xylose, however, there is no such constraint, and the acetyl-P can be used for generating ATP. Table S1. Production of 2-butanol from lactose and xylose in defined SA medium using resting cells of L. lactis and L. brevis. [file 13068_2020_1689_MOESM1_ESM.docx]

# Additional file 1


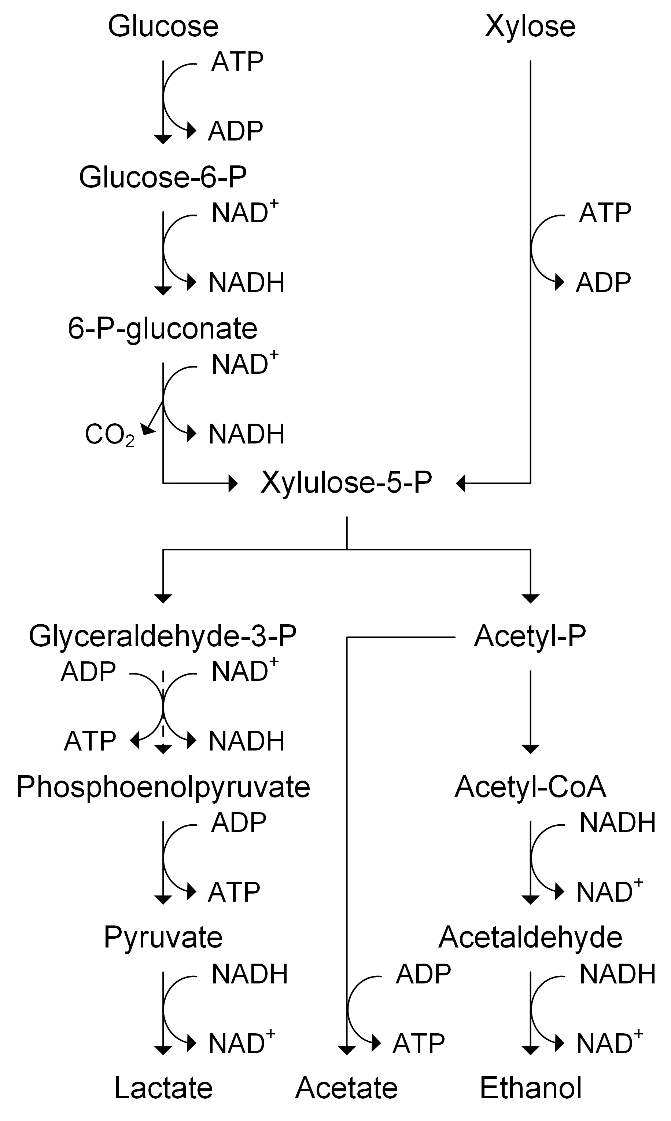


**Figure S1 Overview of glucose and xylose metabolism in *L. brevis*.** On glucose, two NADH are formed in the oxidative pentose phosphate pathway, and these have to be oxidized through ethanol formation from acetyl-CoA. Thus, the Acetyl-P cannot give rise to ATP formation through the action of acetate kinase. On xylose, however, there is no such constraint, and the acetyl-P can be used for generating ATP.

Table S1 Production of 2-butanol from lactose and xylose in defined SA medium using resting cells of *L. lactis* and *L. brevis*.

| Sugar | 2-butanol  mM | Yield^a^  mol/mol |
| --- | --- | --- |
| Glucose | 14.2±0.6 | 0.5±0.02 |
| Lactose + Xylose | 13.0±0.01 | 0.1±0.001 |

To allow lactose utilization in our constructed *L. lactis*, we re-introduced the lactose plasmid pLP712 by transformation. On the mixed carbon, *L. lactis* can utilize only lactose whereas *L. brevis* can utilize only xylose.

Strains were incubated in defined SA medium with glucose or lactose and xylose, 2-butanone, and vitamin B12 for 20 h. Values are average of three independent experiments with standard deviations. ^a^ 2-butanol yield per mol sugar in glucose units, excluding the added 2-butanone.
